# Deciphering Immune Modulation in Chickens Co-Infected with ALV-J and CIAV: A Transcriptomic Approach

**DOI:** 10.3390/microorganisms12122453

**Published:** 2024-11-28

**Authors:** Sheng Chen, Huijuan Xu, Wenxue Li, Yu Nie, Qingmei Xie, Weiguo Chen

**Affiliations:** 1 State Key Laboratory of Swine and Poultry Breeding Industry & Heyuan Branch, Guangdong Provincial Laboratory of Lingnan Modern Agricultural Science and Technology, College of Animal Science, South China Agricultural University, Guangzhou 510642, China; chens@scau.edu.cn (S.C.); sunnyxu20181@outlook.com (H.X.); cs@scau.stu.edu.cn (W.L.); thcscau@163.com (Y.N.); qmx@scau.edu.cn (Q.X.); 2 Guangdong Provincial Key Lab of AgroAnimal Genomics and Molecular Breeding, College of Animal Science, South China Agricultural University, Guangzhou 510642, China; 3 Guangdong Engineering Research Center for Vector Vaccine of Animal Virus, Guangzhou 510642, China

**Keywords:** avian leukosis virus subgroup J (ALV-J), chicken infectious anemia virus (CIAV), co-infection, miRNA–mRNA

## Abstract

Viral co-infections pose significant challenges, causing substantial economic losses worldwide in the poultry industry. Among these, avian lLeukosis virus subgroup J (ALV-J) and chicken infectious anemia virus (CIAV) are particularly concerning, as they frequently lead to co-infections in chickens, further compromising their immune defenses, increasing susceptibility to secondary infections and diminishing vaccine efficacy. While our previous studies have examined the pathogenicity and immunosuppressive effects of these co-infections in vitro and in vivo, the key genes and molecular pathways involved remain largely unexplored. This study investigates the synergistic effects of co-infection with ALV-J and CIAV through comprehensive transcriptome analysis using high-throughput sequencing. We identified 1007 differentially expressed mRNAs (DEmRNAs) and 62 differentially expressed miRNAs (DEmiRNAs) associated with the synergistic activation effects of co-infection, along with 331 DEmRNAs and 62 DEmiRNAs linked to specific activation processes. Notably, the immune suppression observed in co-infected chickens may be influenced by the enhanced utilization of reactive oxygen species (ROS) and oxidative stress pathways, which impact host immune responses. Furthermore, co-infection appears to employ distinct immune evasion strategies through the modulation of rRNA metabolism, differing from single infections. These insights provide a deeper understanding of the molecular mechanisms underlying immune suppression during viral co-infections and help develop targeted therapies and improve disease control in poultry, reducing economic losses.

## 1. Introduction

Immunosuppressive viral diseases weaken chickens’ immune defenses, making them more prone to secondary infections and less responsive to vaccines, which poses a serious challenge to the global poultry industry [1,2]. Studies increasingly indicate that co-infections with immunosuppressive viruses are widespread in poultry and often result in more-severe disease manifestations [3,4]. For instance, our recent findings revealed that superinfection with avian leukosis virus subgroup J (ALV-J) and infectious bursal disease virus (IBDV) exacerbate immunosuppression and increase disease severity in chickens [5]. Similarly, both our research and studies by other groups have highlighted the detrimental effects of co-infection with ALV-J and chicken infectious anemia virus (CIAV) in amplifying immunosuppression, facilitating viral replication, and worsening pathogenic outcomes [6,7,8]. Despite these insights, the specific genes and molecular pathways driving the aggravated disease processes during co-infection remain largely unexplored.

The identification of key differentially expressed genes during co-infection is complicated by the intricate interplay of multiple factors [9,10]. To overcome this challenge, we established a novel approach that analyzes both synergistic and specific gene activation patterns in co-infected hosts. Synergistic activation involves genes or pathways that can be stimulated by either virus individually but exhibit markedly increased expression when both viruses co-occur. In contrast, specific activation refers to genes or pathways that are exclusively induced in the presence of both viruses. These two analytical frameworks offer complementary perspectives, providing deeper insights into the molecular mechanisms underlying the pathogenic processes of co-infection.

In this study, we performed a comprehensive miRNA–mRNA enrichment analysis to reveal both synergistic and specific activation patterns during the co-infection of ALV-J and CIAV. Our results suggest that the observed immunosuppression may be attributed to the intensified activation of reactive oxygen species (ROS) and oxidative stress pathways, which interfere with the host’s immune regulation. Furthermore, compared to individual infections, co-infection appears to leverage unique immune evasion mechanisms by modulating rRNA metabolism. These findings offer novel insights into the molecular mechanisms underlying immune suppression in viral co-infections.

## 2. Materials and Methods

### 2.1. Ethics Statement

Institutional and national guidelines for the use and care of laboratory animals were closely followed. All animal experiments were performed following the guidelines of the South China Agricultural University Animal Care and Use Committee (permit no. SCAU2021b020). The study was performed in positive-pressure high-efficiency particulate air-filtered stainless-steel isolators with an enclosed and ventilated environment, and feed and water were provided ad libitum.

### 2.2. Virus and Animals

ALV-J strain SCAU-HN06 was a generous gift from Professor Liao of the South China Agricultural University. The GD-101 strain of CIAV was stored in our laboratory. One-day-old SPF chicks (White Leghorn), including both hen chicks and cock chicks, were purchased from Xinxing Dahuanong Poultry Eggs Co., Ltd. (Yunfu, China).

### 2.3. Animal Experimental Design and Sample Collection

**Figure 1:**
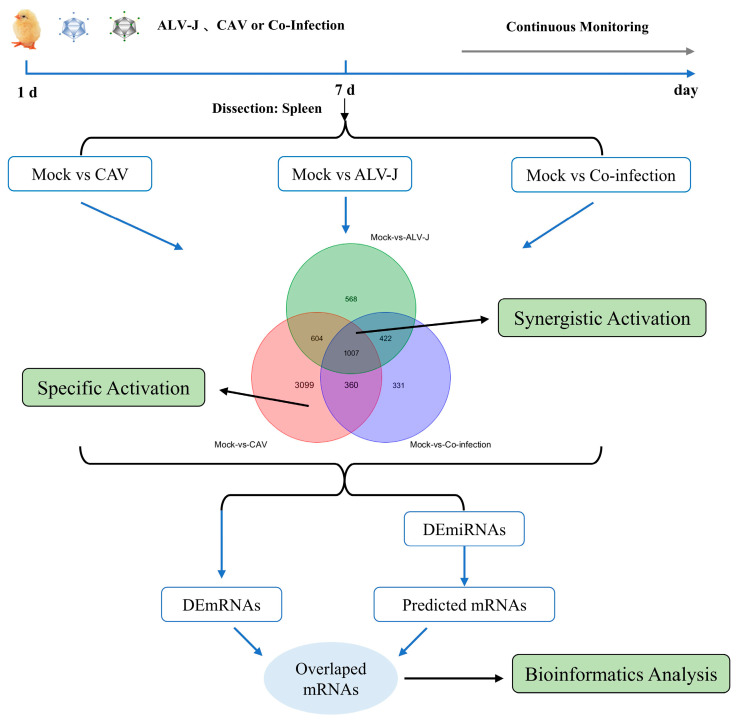
Workflow of bioinformatics analysis.

A total of 160 one-day-old SPF chicks were randomly divided into four groups (40 chicks per group): ALV-J, CIAV, ALV-J+CIAV, and a control group. Each group was housed in separate negative-pressure isolators equipped with filtered air systems. ALV-J mono-infected chickens were inoculated with 10^4.5^ TCID_50_ of the ALV-J strain SCAU-HN06 in 0.2 mL PBS through intraabdominal injection. CIAV mono-infected chickens were inoculated with 1 × 10^6^ copy numbers of the CIAV strain GD-101 in 0.2 mL PBS by leg muscle injection. Co-infected chickens were inoculated with 10^4.5^ TCID_50_ of ALV-J and 1 × 10^6^ copy numbers of CIAV in 0.2 mL PBS. Mock-infected chickens were inoculated with 0.2 mL PBS. At 7 days post-infection, three chicks from each group were randomly selected for the collection of spleens, which were then sent to Genedenovo Biotechnology Co., Ltd. (Guangzhou, China) for full transcriptome high-throughput sequencing. The specific treatments are detailed in Figure 1. 

### 2.4. Differentially Expressed mRNA and miRNA

To identify differentially expressed transcripts across samples or groups, the edgeR package (http://www.bioconductor.org/packages/release/bioc/html/edgeR.html accessed on 26 November 2024) was used. We identified mRNA and lncRNA with a fold change of ≥2 and a false discovery rate (FDR) of <0.05 in a comparison as significant DEGs, and miRNA with a fold change of ≥2 and *p* < 0.05.

### 2.5. MiRNA Target Prediction

For samples, a triad of computational tools—mireap, miRanda, and TargetScan—were harnessed to discern miRNA targets. Information pertaining to miRNA sequences and their respective families was gleaned from the TargetScan online repository (accessible at http://www.targetscan.org/ accessed on 26 November 2024).

### 2.6. Construction of the miRNA–Target Network

Expression correlation between the miRNA and target was evaluated using the Pearson correlation coefficient (PCC). For the multi-group, pairs with a PCC < −0.7 and *p* < 0.05 were selected as negatively co-expressed miRNA–target pairs, and all RNAs were differentially expressed.

### 2.7. Visualization of the miRNA–Target Network

The miRNA–target network was constructed as above, and then visualized using Cytoscape software (v3.6.0) (http://www.cytoscape.org/).

### 2.8. Functional Enrichment Analysis

To assess functional enrichment, the Gene Ontology (GO) Biological Processes term and Kyoto Encyclopedia of Genes and Genomes (KEGG) pathway analyses of mRNAs in the network were conducted. For circRNA, we performed the functional enrichment analysis of source genes to study the main functions of these source genes of circRNAs. GO enrichment analysis provides all GO terms that are significantly enriched in genes compared to the genome background, and filters the genes that correspond to biological functions. Firstly, all genes were mapped to GO terms in the Gene Ontology database (http://www.geneontology.org/), gene numbers were calculated for every term, and significantly enriched GO terms in genes compared to the genome background were defined by a hypergeometric test. Genes usually interact with each other to play roles in certain biological functions. Pathway-based analysis helps to further understand gene biological functions. KEGG is the major public pathway-related database (http://www.kegg.jp/kegg/). Pathway enrichment analysis identified significantly enriched metabolic pathways or signal transduction pathways in genes compared with the whole genome background.

## 3. Results

### 3.1. Identification of Differentially Expressed miRNAs (DEmiRNAs) and mRNAs (DEmRNAs) in the Co-Infection of ALV-J and CIAV

Our previous research results indicated that co-infection with ALV-J and CIAV synergistically causes pathogenicity by enhancing viral replication, resulting in more-severe immunosuppression than infections with either virus alone [6]. However, the precise molecular mechanisms driving this synergistic effect remain unknown. To investigate further, we randomly selected three chickens from each experimental group (ALV-J, CIAV, ALV-J+CIAV, and control) at 7 days post-infection and collected spleen samples for comprehensive transcriptome analysis using high-throughput sequencing.

As illustrated in Figure 1, this strategy was employed to analyze the miRNA–mRNA target network during the co-infection process with ALV-J and CIAV, with an emphasis on both synergistic and specific activations. In addressing synergistic activation, we filtered differentially expressed mRNAs and miRNAs that exhibit synergistic activation for integrative analysis. For specific activation, differentially expressed, characteristically activated RNAs were selected, and a similar gene network analysis was conducted.

**Figure 2:**
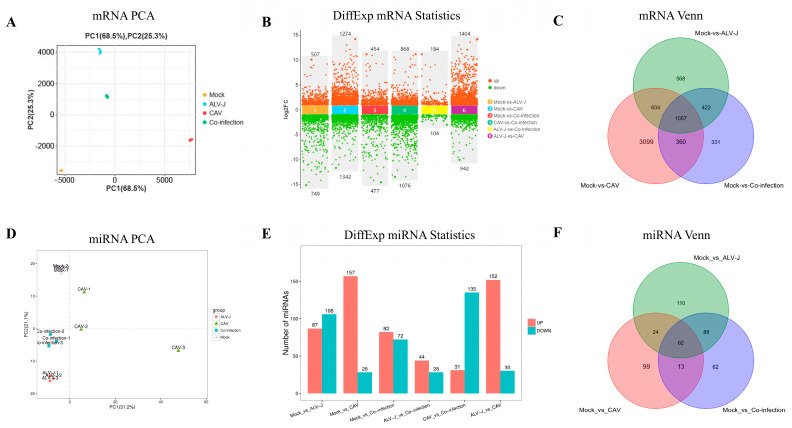
Statistical analysis of differentially expressed RNAs. (**A**) Principal components analysis of mRNAs. (**B**) Differentially expressed mRNAs. (**C**) Venn diagram of mRNAs. (**D**) Principal components analysis of miRNAs. (**E**) Differentially expressed miRNAs. (**F**) Venn diagram of miRNAs.

To ensure high-quality data for analysis, it is crucial to filter raw sequencing data to reduce noise from invalid entries. In this study, we employed FastP for quality control, removing low-quality reads to generate clean data for further processing (Supplementary Table S1). The clean reads were then aligned to the species’ ribosomal database using Bowtie2, with only perfect matches allowed to eliminate ribosomal sequences. Reads that did not align were retained for subsequent transcriptomic analysis. Principal component analysis (PCA) revealed distinct clusters for both single-infection and co-infection groups, confirming that the miRNA and mRNA data obtained are robust and suitable for downstream analyses (Figure 2A,D). 

For the differential expression analysis across different RNA types, we identified significantly differentially expressed mRNAs with an FDR < 0.05 and |log2FC| > 1, and miRNAs with *p* < 0.05 and |log2FC| > 1. Regarding mRNAs, a total of 749 genes were downregulated and 507 were upregulated in the ALV-J group compared to the control group. In the CIAV group, 1342 genes showed downregulation, while 1274 were upregulated. Meanwhile, the co-infection group exhibited 477 downregulated and 454 upregulated genes (Figure 2B and Supplementary Table S2). The corresponding results for differentially expressed miRNAs are illustrated in Figure 2E and Supplementary Table S3.

To identify genes that play a pivotal role during the co-infection process, the strategy delineated above was employed to analyze differentially expressed genes across comparison groups (Figure 2C,F). Among these, there were 1007 mRNAs and 62 miRNAs synergistically activated during co-infection (Supplementary Table S4). Additionally, 331 mRNAs and 62 miRNAs were found to be specifically activated under co-infection conditions (Supplementary Table S5). To further explore the mechanisms behind these synergistic and specific activation patterns, miRNA–mRNA interaction networks were constructed, and functional enrichment analysis was conducted on the overlapping mRNAs.

### 3.2. Biological Functions of DEmRNAs During Co-Infection with Synergistic Activation

**Figure 3:**
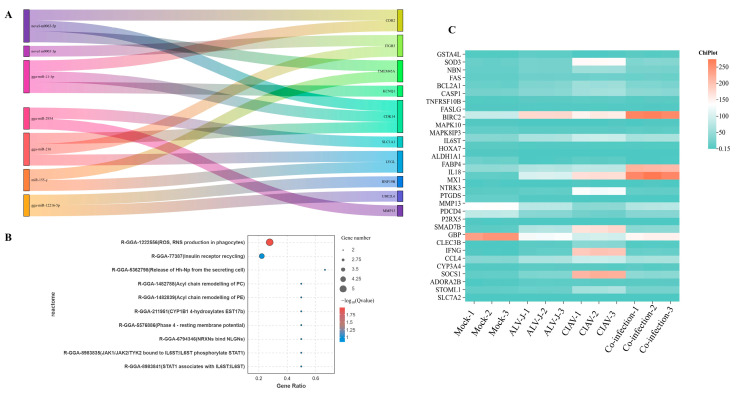
Biological functions analysis of DEmiRNAs and DEmRNAs during co-infection with synergistic activation. (**A**). Sankey diagram of the top 10 miRNA–mRNA pairs with the highest significance. (**B**). Bubble chart of the significantly enriched metabolic processes. (**C**). Heatmap of key genes involved in ROS.

A Sankey diagram was generated to visualize the regulatory interactions between miRNAs and the top 10 differentially expressed mRNAs (Figure 3A). In addition, we explored the metabolic pathways associated with the differentially enriched genes. The analysis revealed that co-infection with ALV-J and CIAV synergistically activates metabolic pathways linked to the production of reactive oxygen species (ROS) and reactive nitrogen species (RNS) in phagocytes (Figure 3B and Supplementary Table S6). Notably, Several genes, such as FASLG, MX1, and GBP, exhibit notable upregulation under co-infection conditions, suggesting a heightened oxidative stress response or immune activation. Additionally, inflammatory markers such as IL18 and IFNG demonstrate differential expression, with increased activity in certain infection states, reflecting their role in modulating inflammation in response to ROS (Figure 3C). 

**Figure 4:**
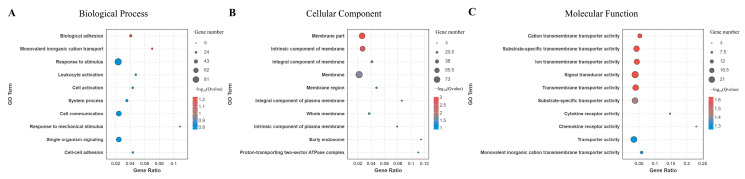
GO analysis of DEmiRNAs and DEmRNAs during co-infection with synergistic activation. (**A**). Biological Process. (**B**). Cellular Component. (**C**). Molecular Function.

GO annotation further identified key functional roles associated with the synergistic activation during co-infection (Supplementary Table S7). The GO analysis indicated that the most enriched biological processes (BPs) involve biological adhesion and the transport of monovalent inorganic cations (Figure 4A). For cellular components (CCs), membrane-related categories, such as membrane parts and intrinsic components of membranes, were highly enriched (Figure 4B). In terms of molecular function (MF), the differentially expressed mRNAs were primarily involved in transmembrane transporter activities, including cation, ion, and substrate-specific transmembrane transport (Figure 4C).

**Figure 5:**
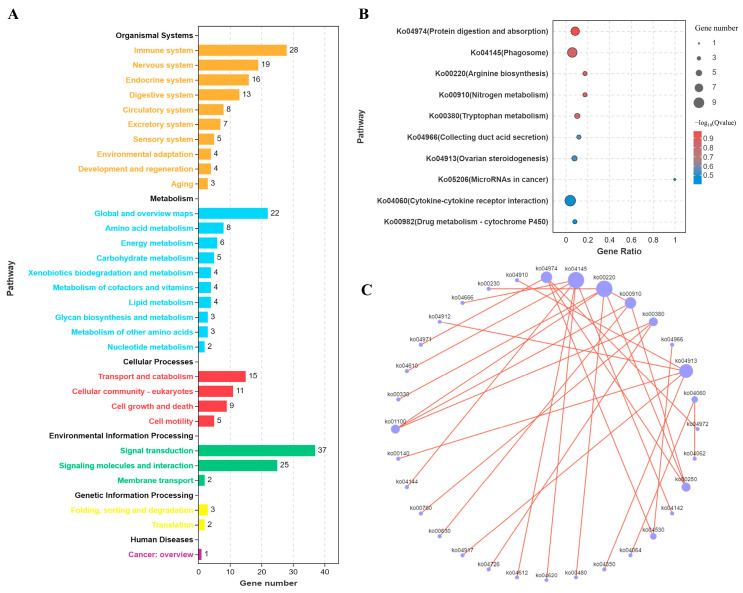
KEGG pathway analysis of DEmiRNAs and DEmRNAs during co-infection with synergistic activation. (**A**). Bar chart of the number of KEGG pathways. (**B**). Bubble chart of KEGG enrichment results. (**C**). Network diagram of KEGG pathway correlations.

In the KEGG pathway enrichment analysis, we first obtained an overview of the KEGG pathways enriched with differentially expressed genes. The results revealed that these genes were primarily concentrated in immune-system-related pathways. Additional enriched pathways involved amino acid metabolism, transport, and degradation, as well as signal transduction processes (Supplementary Table S8 and Figure 5A). Further analysis of specific pathways showed that co-infection with ALV-J and CIAV significantly enhanced processes such as protein digestion, absorption, and phagosome formation (Figure 5B). Pathway correlation analysis identified the phagosome pathway (ko04145) as the most interconnected with other KEGG pathways (Figure 5C). Overall, these findings suggest that ALV-J and CIAV co-infection intensifies host immune responses, with the phagosome pathway playing a particularly prominent role.

### 3.3. Biological Functions of DEmRNAs During Co-Infection with Specific Activation

**Figure 6:**
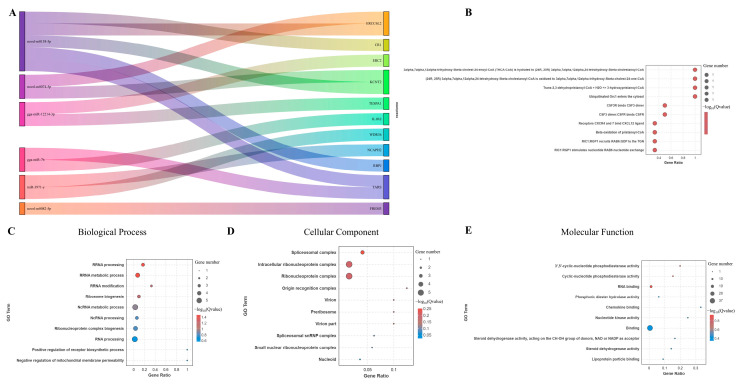
Biological function analysis of DEmiRNAs and DEmRNAs during co-infection with specific activation. (**A**). Sankey diagram of the top 10 miRNA–mRNA pairs with the highest significance. (**B**). Bubble chart of the significantly enriched metabolic processes. (**C**). Biological Process. (**D**). Cellular Component. (**E**). Molecular Function.

Similarly, a Sankey diagram was constructed to depict the regulatory interactions between the top 10 differentially expressed mRNAs and their corresponding miRNA targets (Figure 6A). Additionally, we investigated the metabolic pathways associated with these enriched genes (Supplementary Table S9). The analysis revealed that co-infection with ALV-J and CIAV specifically activated pathways linked to CoA oxidative metabolism (Figure 6B). GO annotation further identified key gene functions triggered during co-infection (Supplementary Table S10). Among the biological processes (BPs), rRNA processing and rRNA metabolic activity were the most significantly enriched (Figure 6C). For cellular components (CCs), the spliceosomal complex, intracellular ribonucleoprotein complex, and ribonucleoprotein complex exhibited the highest enrichment levels (Figure 6D). In terms of molecular function (MF), the differentially expressed mRNAs were predominantly associated with nucleotide phosphodiesterase activity (Figure 6E). 

**Figure 7:**
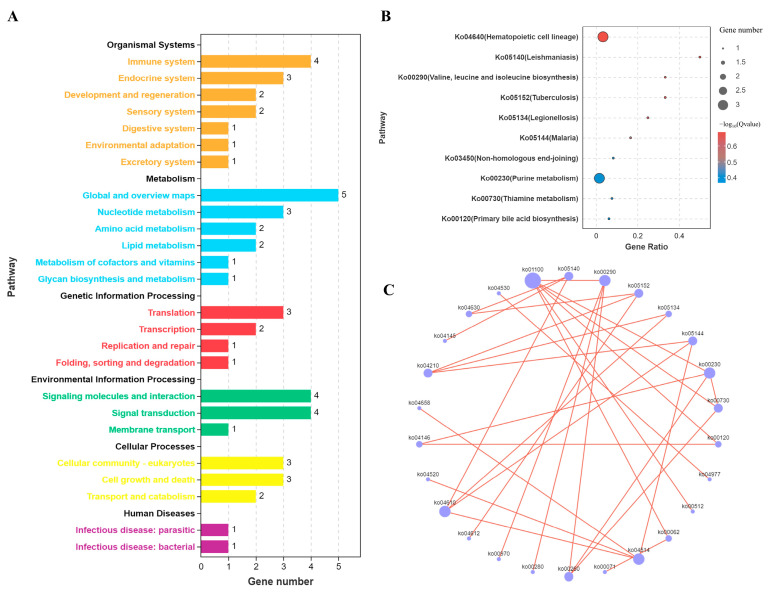
KEGG pathway analysis of DEmiRNAs and DEmRNAs during co-infection with specific activation. (**A**). Bar chart of the number of KEGG pathways. (**B**). Bubble chart of KEGG enrichment results. (**C**). Network diagram of KEGG pathway correlations.

In the KEGG pathway enrichment analysis, we first provided an overview of the KEGG pathways enriched with differentially expressed genes (Supplementary Table S11). The results showed that miRNAs and mRNAs uniquely activated during co-infection were also predominantly enriched in immune system pathways. Other significant pathways included nucleotide metabolism, translation, and signal transduction (Figure 7A). Further examination revealed that co-infection with ALV-J and CIAV strongly activated the hematopoietic cell lineage pathway (Figure 7B). Pathway correlation analysis identified the metabolic pathway (ko01100) as the most interconnected with other KEGG pathways (Figure 7C). These findings suggest that ALV-J and CIAV co-infection selectively enhances host immune responses, with a notable emphasis on metabolic pathways.

## 4. Discussion

Viral co-infections pose significant challenges, causing substantial economic losses worldwide in the poultry industry. Recent studies have demonstrated that co-infection with ALV-J and Marek’s disease virus (MDV) significantly promotes tumor initiation and metastasis, highlighting its critical role in the pathogenesis of avian diseases [11]. Our previous studies investigated the pathogenicity and immunosuppressive effects of ALV-J and CIAV co-infections through both in vitro and in vivo experiments [6]. However, the key genes and molecular pathways driving these effects remain unclear. In this study, we collect spleen samples for comprehensive transcriptome analysis using high-throughput sequencing. To uncover the synergistic and specific activation mechanisms of co-infection, we analyzed the sequencing data, predicted miRNA–mRNA interactions, and performed an enrichment analysis on the overlapping mRNAs. The observed synergistic activation during co-infection implies that while individual viruses can initiate these functions, their presence together leads to a significant enhancement. Notably, co-infection significantly influences metabolic pathways associated with reactive oxygen species (ROS) and reactive nitrogen species (RNS) production in macrophages, activating the differential expression of related metabolic genes. This finding suggests that the observed synergistic activation in ALV-J and CIAV co-infection may play a role in immune response regulation through mechanisms involving oxidative stress.

Viruses exploit ROS and oxidative metabolic pathways to manipulate host immune responses, significantly influencing infection dynamics [12]. ROS are generated as by-products of oxidative metabolism in immune cells and serve as signaling molecules that modulate immune functions [13]. ROS are generated as by-products of oxidative metabolism in immune cells and serve as signaling molecules that modulate immune functions [14,15]. Numerous studies indicate that increased ROS levels can regulate immune responses by activating key signaling pathways critical for both innate and adaptive immunity. For instance, viruses like hepatitis B virus (HBV) and other pathogens stimulate ROS production through mechanisms such as mitochondrial dysfunction and p53 activation, thereby affecting immune cell activity and survival [16]. HBV’s manipulation of ROS pathways is associated with persistent infections, as it alters the effector functions of immune cells, including cytotoxicity and cytokine production, which are vital during infections and cancer development [17]. Additionally, CIAV enhances intracellular ROS through the apoptin protein, leading to the aggregation of the mitochondrial membrane protein Tom20 and ultimately triggering pyroptosis [18]. However, research on the role of ROS in ALV-J infection remains lacking. Furthermore, oxidative metabolism influences the functionality of key immune cells, such as macrophages and natural killer (NK) cells, which depend on ROS for activities like phagocytosis and cytotoxic responses [19,20]. The interplay between ROS and immune pathways, including the metabolic networks associated with phagosomal processes, underscores the complexity of immune regulation during viral co-infections involving ALV-J and CIAV.

The specific activation observed during co-infection pertains to functions that are not induced by either virus independently but are significantly enhanced when both viruses are present, underscoring the unique effects of co-infection. Our results indicate that the GO terms enriched by this specific activation include biological processes related to rRNA metabolism, cellular components associated with ribonucleoprotein complexes, and molecular functions involving RNA binding, highlighting the importance of rRNA metabolism in the context of co-infection.

Viral infections can strategically manipulate host ribosomal RNA (rRNA) metabolism to influence immune responses, promoting both viral replication and evasion of the host immune system [21,22]. Viral infections can strategically manipulate host ribosomal RNA (rRNA) metabolism to influence immune responses, promoting both viral replication and evasion of the host immune system [23,24]. This increased rRNA production supports the cellular machinery required for synthesizing viral proteins, while simultaneously compromising the host’s capacity to mount effective immune responses by reallocating resources toward ribosome production rather than essential immune signaling molecules, such as interferons [25,26]. To date, no prior research has established a connection between immune regulation and the modulation of rRNA metabolism in the context of ALV-J and CIAV infections. Our results suggest that co-infection with ALV-J and CIAV may specifically activate this process, thereby regulating immune responses through modifications in rRNA metabolism. This finding offers new insights into the mechanisms underlying the severe immunosuppression linked to these co-infections and proposes a novel perspective for understanding how viral interference with ribosomal functions affects host immunity.

## 5. Conclusions

In conclusion, we conducted a thorough miRNA–mRNA enrichment analysis that clarified both synergistic and specific activations during the co-infection of ALV-J and CIAV. Our results suggest that the immune suppression observed in co-infected subjects may be influenced by increased utilization of ROS and oxidative stress pathways, which affect the host’s immune responses. Furthermore, co-infection appears to adopt distinct immune evasion mechanisms through the modulation of rRNA metabolism, in contrast to single infections. These findings offer novel insights into the molecular underpinnings of immune suppression during viral co-infection and may help to develop targeted therapies and improve disease control in poultry, reducing economic losses.

## Data Availability

All the data generated or analyzed in this study are included in this paper.

## Supplementary Materials

The following supporting information can be downloaded at: https://www.mdpi.com/article/10.3390/microorganisms12122453/s1; Table S1, Clean Reads; Table S2, all.diff.mRNA; Table S3, all.diff.miRNA; Table S4, Synergistic Activation_mRNA_miRNA; Table S5, Specific Activation_mRNA_miRNA; Table S6, Synergistic Activation_Reactome; Table S7, Synergistic Activation_GO; Table S8, Synergistic Activation_KEGG; Table S9, Specific Activation_Reactome; Table S10, Specific Activation_GO; Table S11, Specific Activation_KEGG.

## References

[1] Hoerr F.J. (2010). Clinical aspects of immunosuppression in poultry. Avian Dis..

[2] Gimeno I.M., Schat K.A. (2018). Virus-Induced Immunosuppression in Chickens. Avian Dis..

[3] Zheng L.-P., Teng M., Li G.-X., Zhang W.-K., Wang W.-D., Liu J.-L., Li L.-Y., Yao Y., Nair V., Luo J. (2022). Current Epidemiology and Co-Infections of Avian Immunosuppressive and Neoplastic Diseases in Chicken Flocks in Central China. Viruses.

[4] Dong X., Ju S., Zhao P., Li Y., Meng F., Sun P., Cui Z. (2014). Synergetic effects of subgroup J avian leukosis virus and reticuloendotheliosis virus co-infection on growth retardation and immunosuppression in SPF chickens. Vet. Microbiol..

[5] Chen W., Chen S., Nie Y., Li W., Li H., Zhang X., Chen F., Xie Q. (2022). Synergistic Immunosuppression of Avian Leukosis Virus Subgroup J and Infectious Bursal Disease Virus Is Responsible for Enhanced Pathogenicity. Viruses.

[6] Xu H., Li W., Nie Y., Chen S., Li H., Zhang X., Xie Q., Chen W. (2024). Synergy of Subgroup J Avian Leukosis Virus and Chicken Infectious Anemia Virus Enhances the Pathogenicity in Chickens. Microorganisms.

[7] Zhang J., Ma L., Li T., Li L., Kan Q., Yao X., Xie Q., Wan Z., Shao H., Qin A. (2021). Synergistic pathogenesis of chicken infectious anemia virus and J subgroup of avian leukosis virus. Poult. Sci..

[8] Ma L., Zhang Y., Wang J., Wang Y., Chang S., Zhao P. (2024). Synergistic pathogenicity of vertically transmitted chicken infectious anemia virus and avian leukosis virus subgroup J coinfection in chickens. Poult. Sci..

[9] Paget C., Trottein F. (2019). Mechanisms of Bacterial Superinfection Post-influenza: A Role for Unconventional T Cells. Front. Immunol..

[10] Semrau A., Wibbelt G., Hilbe M., Lieckfeldt D., Hermes R., Müller K.E., Heckert H.P., Hoyer M.J., Frölich K. (2008). Experimental superinfection of a Lesser Malayan mousedeer (*Tragulus javanicus*) persistently infected with bovine viral diarrhea virus. J. Zoo Wildl. Med..

[11] Zhou J., Zhou D., Zhang Q., Zhang X., Liu X., Ding L., Wen J., Xu X., Cheng Z. (2024). DCLK1 mediated cooperative acceleration of EMT by avian leukosis virus subgroup J and Marek’s disease virus via the Wnt/β-catenin pathway promotes tumor metastasis. J. Virol..

[12] He M., Wang M., Xu T., Zhang M., Dai H., Wang C., Ding D., Zhong Z. (2023). Reactive oxygen species-powered cancer immunotherapy: Current status and challenges. J. Control. Release.

[13] Sahoo B.M., Banik B.K., Borah P., Jain A. (2022). Reactive Oxygen Species (ROS): Key Components in Cancer Therapies. Anti-Cancer Agents Med. Chem..

[14] Wang L., Cao Z., Wang Z., Guo J., Wen J. (2022). Reactive oxygen species associated immunoregulation post influenza virus infection. Front. Immunol..

[15] Foo J., Bellot G., Pervaiz S., Alonso S. (2022). Mitochondria-mediated oxidative stress during viral infection. Trends Microbiol..

[16] Kim S., Park J., Han J., Jang K.L. (2024). Hepatitis B Virus X Protein Induces Reactive Oxygen Species Generation via Activation of p53 in Human Hepatoma Cells. Biomolecules.

[17] Jeong Y., Han J., Jang K.L. (2024). Reactive Oxygen Species Induction by Hepatitis B Virus: Implications for Viral Replication in p53-Positive Human Hepatoma Cells. Int. J. Mol. Sci..

[18] Liu Z., Li Y., Zhu Y., Li N., Li W., Shang C., Song G., Li S., Cong J., Li T. (2022). Apoptin induces pyroptosis of colorectal cancer cells via the GSDME-dependent pathway. Int. J. Biol. Sci..

[19] Ganeshan K., Chawla A. (2014). Metabolic regulation of immune responses. Annu. Rev. Immunol..

[20] Van den Bossche J., Baardman J., Otto N.A., van der Velden S., Neele A.E., van den Berg S.M., Luque-Martin R., Chen H.J., Boshuizen M.C., Ahmed M. (2016). Mitochondrial Dysfunction Prevents Repolarization of Inflammatory Macrophages. Cell Rep..

[21] Li S. (2019). Regulation of Ribosomal Proteins on Viral Infection. Cells.

[22] Rawlinson S.M., Zhao T., Ardipradja K., Zhang Y., Veugelers P.F., Harper J.A., David C.T., Sundaramoorthy V., Moseley G.W. (2023). Henipaviruses and lyssaviruses target nucleolar treacle protein and regulate ribosomal RNA synthesis. Traffic.

[23] Bianco C., Mohr I. (2019). Ribosome biogenesis restricts innate immune responses to virus infection and DNA. Elife.

[24] Lee A.J., Feng E., Chew M.V., Balint E., Poznanski S.M., Giles E., Zhang A., Marzok A., Revill S.D., Vahedi F. (2022). Type I interferon regulates proteolysis by macrophages to prevent immunopathology following viral infection. PLoS Pathog..

[25] Insua J.L., Llobet E., Moranta D., Pérez-Gutiérrez C., Tomás A., Garmendia J., Bengoechea J.A. (2013). Modeling *Klebsiella pneumoniae* pathogenesis by infection of the wax moth *Galleria mellonella*. Infect. Immun..

[26] Beura L.K., Dinh P.X., Osorio F.A., Pattnaik A.K. (2011). Cellular poly(c) binding proteins 1 and 2 interact with porcine reproductive and respiratory syndrome virus nonstructural protein 1β and support viral replication. J. Virol..

